# Developing a leadership pipeline: the Cleveland Clinic experience

**DOI:** 10.1007/s40037-014-0135-y

**Published:** 2014-08-01

**Authors:** Caryl A. Hess, Christina Barss, James K. Stoller

**Affiliations:** 1 Education Institute, Cleveland Clinic, 9500 Euclid Avenue, NA22, Cleveland, OH 44195 USA; 2 Department of Medicine, Cleveland Clinic Lerner College of Medicine, Cleveland Clinic, 9500 Euclid Avenue, NA22, Cleveland, OH 44195 USA; 3 Cleveland Clinic Academy, Cleveland Clinic, 9500 Euclid Avenue, NA22, Cleveland, OH 44195 USA

**Keywords:** Leadership, Development, Pipeline, Healthcare, Physician

## Abstract

The complexity of health care requires excellent leadership to address the challenges of access, quality, and cost of care. Because competencies to lead differ from clinical or research skills, there is a compelling need to develop leaders and create a talent pipeline, perhaps especially in physician-led organizations like Cleveland Clinic. In this context, we previously reported on a cohort-based physician leadership development course called Leading in Health Care and, in the current report, detail an expanded health care leadership development programme called the Cleveland Clinic Academy (CCA). CCA consists of a broad suite of offerings, including cohort-based learning and ‘a la carte’ half- or full-day courses addressing specific competencies to manage and to lead. Academy attendance is optional and is available to all physicians, nurses, and administrators with the requisite experience. Course selection is guided by competency matrices which map leadership competencies to specific courses. As of December 2012, a total of 285 course sessions have been offered to 6,050 attendees with uniformly high ratings of course quality and impact. During the past 10 years, Cleveland Clinic’s leadership and management curriculum has successfully created a pipeline of health care leaders to fill executive positions, search committees, board openings, and various other organizational leadership positions. Health care leadership can be taught and learned.

## Introduction

Health care institutions are currently challenged by pressing issues regarding universal coverage, the cost of providing care, and the quality of care [1]. In this context, great leadership in health care is urgently needed and health care leaders must have the requisite competencies to navigate increasingly complex environments and organizations [2–9].

Despite the profound need within health care and the considerable attention that has been given to developing leaders in other business sectors [10], attention within health care is only recently emerging [2, 8, 11–19]. For example, in a recent systematic review of leadership training programmes in academic medical centres, Strauss et al. [6] identified only 10 studies that met inclusion criteria and concluded that there was a ‘remarkable paucity of literature that presents the benefits of such leadership training.’ In an earlier review of available programmes to develop health care leaders, Stoller [2] further described the early experience with a Cleveland Clinic leadership development programme (called Leading in Health Care [13]). Since launching that course, the leadership development programme at Cleveland Clinic has expanded considerably, with the formation of the Cleveland Clinic Academy (CCA) in 2006. In extending available reports [6], the current paper describes the CCA—the rationale for forming CCA, the essential leadership competencies that inform the CCA curriculum, practical aspects of developing and delivering the curriculum, lessons learned, and available performance metrics.

### The rationale for leadership development at Cleveland Clinic

The need for leadership development for physicians, nurses, and administrators at the Cleveland Clinic rests in the size (~42,000 employees, ~3,200 faculty), complexity, its culture and history as a physician-led organization [16, 20], and the widely recognized challenges to US health care of providing high quality, broadly accessible, and affordable care [1, 8].

In September 2006, the Clinic launched the CCA as an integrated programme to help develop a pipeline of competent physician, nurse, and administrative leaders. The goals of CCA are to offer a leadership and management curriculum for internal health care providers and to extend the existing leadership development offerings [13] to a broad institutional audience, by presenting multiple courses as half-day or full-day ‘a la carte’ sessions.

In describing CCA, the sections below discuss the development of the curriculum followed by a description of the scope and impact of CCA to date.

## Methods

### Developing the curriculum for CCA: leadership and management competencies for health care

The design of the CCA curriculum recognized that the competencies to lead in health care differ from those needed to be an effective clinician or scientist [21]. Specifically, courses in teamwork and teambuilding are deemed critically important. Paradoxically, although teamwork has been shown to enhance clinical outcomes [22, 23], the current process of selecting and training physicians develops ‘heroic lone healers,’ often blunting physicians’ reflexes and skills to collaborate [3–5, 24–26]. Similarly, emotional intelligence has been widely viewed [28] as an essential characteristic for effective leadership and can be taught [29].

Because competency training must be context-specific [28–31], CCA was designed based on our review of the available literature regarding leadership competencies for health care [2, 32–38]. Focus groups [39], literature review, and interviews with senior health care leaders [38, 39] suggested 16 broad, essential health care leadership competencies (Table 1). In keeping with the important distinction between leading and managing [40], the CCA curriculum was segmented into two tracks—leadership and management. Course objectives were mapped to the competencies and a matrix was created to help prospective CCA attendees identify which courses would address which competencies [41].

**Table 1:** Competencies needed to lead in health care

Competency	Component skills
Emotional intelligence	Self-awareness and regulation of emotions, self-motivation, awareness of others’ emotional states, and proficiency in managing relationships [27]
Professionalism	How we conduct ourselves as caregivers in our interactions with patients and society
Change management	Knowledge of change models, personal change readiness and avidity, timeliness of meeting deadlines
Communication	Teambuilding, active listening skills, negotiation, conflict resolution, giving feedback, awareness of Clinic resources, skills for interacting with the media
Commitment to lifelong learning	Openness to learning and trying new ideas and practices
Commitment to deliver observable results	Actively engaging in the planning, discussion and implementation phases of the project
Finance	Implementing a strategic plan, overall competence, reading a financial statement, developing a business plan, managing a budget, knowledge of reimbursement and insurance, creatively managing resources and people
Regulatory environment	Knowledge of resources for quality improvement, knowledge of JCAHO, CMS, etc., regulations as pertains to the individual’s area
Marketing	Awareness of marketing resources, ability to develop a marketing plan
Recruiting and hiring	Awareness of resources for recruiting and hiring, sensitivity and attention to diversity and legal issues, identifying talent
Awareness of technology	Awareness of leaders and resources (‘how things get done’), openness to learning new technologies and implementing new techniques in the workplace
Process assessment and management	Knowledge of resources for process improvement, knowledge of process improvement strategies
Philanthropy and development	Knowledge of resources for philanthropy and development, develop skills to solicit and cultivate potential donors
Medicolegal issues	Awareness of Clinic legal issues, knowledge of strategies to avoid medical malpractice, skill to manage a malpractice claim
Managing physicians	Ability to recognize the physician in trouble, ability to manage the impaired physician and to motivate physicians
Clinic awareness	Knowledge of Clinic leadership structures, knowledge of success metrics, informed about community service and volunteerism opportunities, knowledge of privileging issues and Clinic bylaws, personal collegiality

After [2], with permission

### Practical features: curriculum development, assessment, faculty and announcing CCA

Specific course ideas originated from many sources, including CCA leadership and clinical caregivers. At times, a physician, nurse, or administrator, or a cross-functional team might contact CCA leadership to present a course idea. Proposals that are deemed robust on full discussion and review are developed more fully and the course is trialled.

Awareness of CCA is promoted through several internal communication strategies: a website on the institutional intranet, posting of course catalogues several times a year, occasional personal communications from CCA leadership, and general announcements of CCA activity (e.g., during new staff orientation sessions, etc.). Also, because attending CCA courses automatically populates each staff member’s annual performance assessment (called the annual professional review or APR [41]), there is special attention to CCA attendance at the time of annual re-appointments.

Because CCA was designed to assure broad availability of courses within the Clinic, eligibility requirements are liberal; courses are available to Clinic faculty, physicians-in-training, nurses, and administrators with a Master’s degree or with >3 years of supervisory experience.

Several processes have been implemented to assess the quality and responsiveness of the curriculum to learners’ goals: (1) faculty are evaluated through attendee feedback, (2) all courses carry continuing medical education (CME) certification with an associated review process, (3) arrangements with several local business schools allow classroom contact hours to transfer to local universities for credit toward MBA degrees, requiring additional review of curricula, and (4) attendance is acknowledged and automatically uploaded to the staff APR report [41].

Faculty for CCA are mostly Clinic physicians, research scientists, nurses, and administrators with requisite interests, competencies and experience to teach the relevant content. To assure relevance to the health care audience, some courses are designed to feature faculty dyads consisting of a Clinic faculty content expert paired with an external faculty member, largely from business schools.

Because early surveys suggested that CME credits would enhance attendance, CME credits are provided for all CCA courses at no cost to the participant.

In the context that course evaluations are important for curriculum success [42], ongoing course evaluations currently take two forms. The first is a composite course evaluation at the end of each class. The second structured assessment of each course is the CME survey, which is completed online within 7 days following the course and which is required to receive CME credits.

## Results

Altogether, 285 CCA course sessions were offered between September 2006 and December 2012. The number of annual courses has steadily increased, with 54 total courses offered in 2012. Some courses were repeated and others were new, typically in a 2:1 ratio (repeat:new). In 2012, the CCA faculty consisted of 83 internal faculty and 13 external faculty members.

Between 2006 and 2012, course attendance steadily increased with 6,050 attendee-days and 3,603 total unique attendees through December 2012. Attendees have included staff (*N* = 2,440), nurses (*N* = 551), and other caregivers (*N* = 3,059).

Overall satisfaction with CCA courses has been consistently high and has progressively risen over 6 years. Using a Likert scale of 1–5 (5 = highest rating), course participants have consistently expressed high levels of satisfaction (i.e., mean rating of 4.8 in 2011).

Between June 2009 and December 2012, 9,154 CME credit hours were awarded to 2,163 CCA attendees. In 2012 alone, 1,073 attendees claimed 4,922 h of CME credit.

As of December 2012, 74 Clinic staff physicians, nurses, and administrators accepted this contact hour transfer to pursue advanced business degrees and another 135 Clinic employees actually registered for an onsite MBA programme.

## Discussion

In the context that leadership development is needed but that published experience of courses to develop health care leaders is sparse [6], the current report describes a 6-year experience with the CCA. To our knowledge, this experience represents the largest attended leadership development programme in a health care institution reported to date. Key findings from this experience are that the CCA has been well-attended and highly rated by attendees and that it aligns with an institutional strategy to develop leadership bench strength, enhance system integration, and to support global initiatives.

To align with the strategy of system integration, the longitudinal cohort-based course within CCA (i.e., the Leading in Health Care course [13]) includes leaders from community hospitals within the Cleveland Clinic Health System (CCHS) and staff physicians from Cleveland Clinic Florida. Attendance by these CCHS caregivers promotes side-by-side learning and interaction with main campus Clinic colleagues. Networking among colleagues (who may have met initially in the course) and project-based teamwork are valued course impacts. Our experience suggests that such networking contributes to organizational cohesiveness, synergy, and innovation.

As previously reported [13], ideas that have been generated by course participant teams are developed into business plans, of which 61 % have had organizational impact. Though not yet formally assessed but consistent with prior observations by Korschun et al. [43], we believe that CCA attendance also enhances organizational engagement.

As the Cleveland Clinic expands globally to develop the Cleveland Clinic Abu Dhabi and to manage Sheikh Khalifa Medical City in Abu Dhabi, leadership training is also offered to emerging leaders in these hospitals. For example, such leaders attend the Samson Global Leadership Academy, which is a 2-week residential executive education leadership development programme [44, 45].

Also, because leadership development is critical for trainees [2–4], programmes have been designed that are specifically directed to graduate medical trainees. For example, a 2-day chief residents workshop in leadership is offered yearly in July. The curriculum for the workshop is based on the CCA experience, with several faculty and sessions offered in both programmes (e.g., emotional intelligence, teambuilding, change management).

In the context that this is, to our knowledge, the largest reported experience with a health care institution-based leadership development course, several shortcomings of the current study warrant comment. First, our observations are descriptive and lack a control group. Also, the findings are based on experience in a single institution and so require replication in other settings to ensure generalizability. Third, available outcome measures were limited to attendees’ subjective ratings. More significant outcomes, such as the leadership progression of CCA participants, observers’ assessments of the leadership qualities of CCA attendees, review of attendees’ accomplishments after course completion, etc., will enhance the assessment and are the subject of ongoing inquiry.

In summary, the CCA represents a novel health care institution-based programme that has been designed to help develop a leadership pipeline for Cleveland Clinic. Ongoing study will help further clarify the impact of the programme, which has shown early promise. Careful alignment of leadership development with organizational goals, as well as the opportunity to make further scholarly contributions to the sparsely studied area of health care provider leadership development, represent important future opportunities.
